# Multi antenna structure assisted by metasurface concept providing circular polarization for 5G millimeter wave applications

**DOI:** 10.1038/s41598-025-02208-3

**Published:** 2025-05-21

**Authors:** Ayman A. Althuwayb, Esraa Mousa Ali, Mohammad Alibakhshikenari, Bal S. Virdee, Nasr Rashid, Khaled Kaaniche, Ahmed Ben Atitallah, Osama I. Elhamrawy, Francisco Falcone

**Affiliations:** 1 Electrical Engineering Department, College of Engineering, Jouf University, 72388 Sakaka, Aljouf Saudi Arabia; 2 Communications and Computer Engineering Department, Al-Ahliyya Amman University, Amman, 19111 Jordan; 3 Department of Electrical and Electronics Engineering, Dogus University, 34775 Umraniye, Istanbul, Türkiye; 4 Electronics Engineering Department, University of Rome “Tor Vergata”, 00133 Rome, Italy; 5 Center for Communications Technology, School of Computing & Digital Media, London Metropolitan University, London, N7 8DB UK; 6 Institute of Smart Cities, Department of Electric, Electronic and Communication Engineering, Public University of Navarre, 31006 Pamplona, Spain

**Keywords:** Multi-antenna structure, Circularly polarized (CP), Metasurface (MTS), 5G millimeter-waves (mm-waves), Electrical and electronic engineering, Electronics, photonics and device physics

## Abstract

This This paper presents a circularly polarized multi-antenna structure designed for 5G millimeter-wave applications. The structure is based on circular patch radiators, each enhanced with metasurface (MTS) characteristics through the integration of multi-split ring slots. Each radiating element is enclosed within a decoupling wall constructed from a microstrip transmission line, which features both wide (capacitive) and thin (inductive) impedance profiles. The antennas are excited from below using metallic pins, which connect to the radiators through via-holes stemming from coplanar waveguide ports on the ground plane. Experimental results demonstrate a wide bandwidth from 25.6 to 29.7 GHz, corresponding to a fractional bandwidth of 14.82%. Additionally, the antenna exhibits stable radiation patterns, with an average gain of 2.7 dBi and a radiation efficiency of 57%. Using a single radiator configuration, a 3 × 3 antenna array was implemented. In this design, electromagnetic coupling between adjacent radiators is significantly reduced. The resulting array, measuring 20 × 20 × 0.32 mm^3^, achieves excellent performance across a wide frequency range from 24 to 31 GHz, corresponding to a bandwidth of 25.45%. Key metrics include an average isolation between radiating elements exceeding 17 dB and an average gain and radiation efficiency of 9.0 dBi and 91.5%, respectively.

## Introduction

Wireless communication has evolved significantly over the decades, driven by an ever-increasing demand for higher data rates and lower latency ^1–4^. This trend continues to escalate due to rising consumer expectations and the growth of data-intensive applications such as Virtual Reality (VR), Augmented Reality (AR), and the Internet of Things (IoT) ^1,2^. To meet these evolving requirements, wireless networks must deliver enhanced Quality of Service (QoS). At the forefront of these advancements is the fifth-generation (5G) New Radio technology ^2–6^. With limited bandwidth in the microwave spectrum, 5G systems are adopting millimeter-wave (mm-wave) and sub-terahertz (THz) frequencies, ranging from 24 to 300 GHz, to accommodate the need for high data rates and low latency ^1–6^. This shift is revolutionizing industries, enabling applications such as smart homes, factories, virtual environments, telemedicine, and automotive technologies ^7^. A detailed overview of 5G’s broad application potential is illustrated in ^8^, while ^5^ provides a comprehensive view of the mm-wave and sub-THz spectrums allocated for 5G deployments.

Although 5G offers a significant leap in performance, improving data rates, latency, capacity, and reliability, researchers are already exploring Beyond 5G (B5G) technologies to achieve even greater advancements ^9^. Current 5G networks support data rates of one to a few gigabits per second (Gbps), but the next generation aims for hundreds of Gbps or even terabits per second (Tbps) with latency below 1 ms (ms) ^10^. Meeting these goals requires overcoming significant challenges, particularly the need to expand the usable radio spectrum ^2,4^. While 5G focuses on mm-wave bands around 60 GHz, B5G efforts are pushing the boundaries toward broader frequency ranges ^11^.

In this context, the development of the sixth generation (6G) of mobile communication has become a focal point of interest for academia and industry. 6G aims to deliver access rates 10 to 100 times higher than 5G, with reduced access delays and improved coverage. Achieving these goals will require innovations in antenna and radio frequency (RF) systems, materials, and manufacturing processes ^12^. Specifically, 6G will likely extend the use of frequency bands beyond mm-wave, incorporating Ka-band and Terahertz (THz) frequencies to enable global coverage and unprecedented performance ^12^.

Antenna technology plays a pivotal role in realizing these advancements. The unique challenges posed by mm-wave and sub-THz frequencies, such as high path losses and limited coverage, demand the development of high-gain, wideband antennas. Patch antennas, favored for their simplicity and cost-effectiveness, require performance enhancements in gain and bandwidth to meet these challenges. Techniques such as superstrate loading have shown promise but may compromise compactness and mechanical stability ^13–16^. Similarly, stacking multiple patch substrates can enhance bandwidth and gain but introduces complexity and size constraints ^17,18^. Dielectric resonator antennas (DRAs) offer an alternative solution by mitigating ohmic losses, although they too face design challenges ^19,20^.

Metasurfaces, known for their ability to manipulate electromagnetic waves, have been integrated into antenna designs to achieve high gain and wideband performance ^21^. However, challenges such as fabrication complexity and gaps between the metasurface and radiator persist ^22^. Recent innovations involve single-layer printed antennas with metasurface-based parasitic elements, though these designs often suffer from low gain and limited bandwidth ^23,24^.

Multiple-input multiple-output (MIMO) antennas are also crucial for enhancing data rates, capacity, and spectral efficiency. However, many existing mm-wave MIMO antennas are linearly polarized (LP), which limits propagation direction and increases susceptibility to multipath interference ^25,26^. Circularly polarized (CP) antennas, which radiate in two orthogonal directions with equal signal strength, offer improved flexibility and immunity to multipath effects, making them ideal for mm-wave and sub-THz applications ^27^. Despite these advantages, CP MIMO antenna designs face challenges related to small physical size and connector constraints at high frequencies ^28^. Current solutions, such as Fabry–Perot CP MIMO antennas, achieve high gain and isolation but are hindered by complex designs and large profiles ^29^. Similarly, dual-band CP MIMO antennas designed for 5G base stations offer limited bandwidth ^30^, underscoring the need for compact, low-profile CP antennas that operate across sub-THz frequencies.

This paper proposes a novel circularly polarized metasurface multi-antenna structure for 5G mm-wave applications. Fabricated on a single layer of Rogers RT5880 substrate with a thickness of 0.6 mm, the design features circular patch radiators loaded with multi-split ring slots to achieve metasurface characteristics. These radiators are integrated within a square-shaped microstrip transmission line, incorporating periodic wide (capacitive) and thin (inductive) impedance profiles. The antennas are excited using metallic pins that connect the radiators to coplanar waveguide (CPW) ports through via-holes in the ground plane. A 3 × 3 antenna array, derived from the single radiator design, significantly mitigates electromagnetic coupling between adjacent radiators, enhancing overall performance.

### Single-element single-layered antenna design

This section describes the design of the proposed wideband single-element CP antenna, which will serve as the basis for the antenna array.

#### Geometry of radiating element

Among traditional methods, using circular patches as radiating elements is a simple and effective approach to generate circularly polarized (CP) radiation. The patch diameter is typically chosen to be about half of the effective wavelength at the desired resonant frequency. To ensure impedance matching, the antenna is excited through a coaxial feed connected to the patch’s center via the ground plane. The initial diameter (*d*) of the circular patch is given by ^13^:

1 $$d=\frac{c}{2f\sqrt{{\varepsilon }_{eff}}}$$

where *c* is the speed of electromagnetic waves and ${\varepsilon }_{eff}$ is the effective dielectric constant of the substrate.

**Fig. 1:**
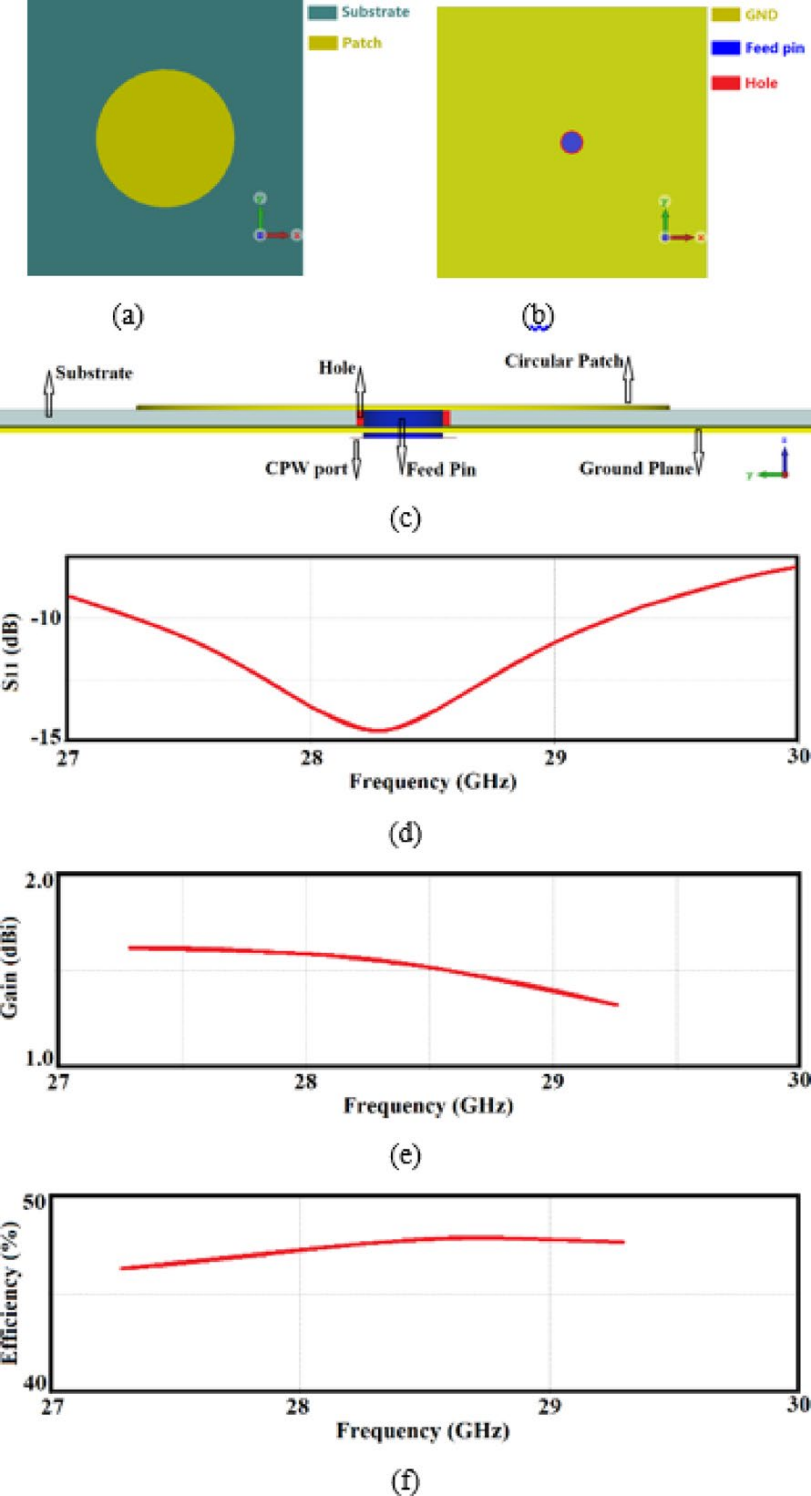
Proposed single-element antenna, (**a**) top-side view, (**b**) bottom view, (**c**) cross-section view, (**d**) reflection coefficient response (S_11_ < -10 dB), (**e**) gain, and (**f**) radiation efficiency.

The patch diameter was carefully adjusted to ensure that the phase and magnitude of the two orthogonal modes produce circularly polarized (CP) radiation. In this design, the patch diameter was set to 4 mm. The proposed single-element CP antenna is illustrated schematically in Fig. 1a–c. It consists of a conductive radiator, 0.035 mm thick, etched on the top side of a single layer of Rogers RT5880 dielectric substrate with a thickness of 0.6 mm, a relative permittivity ${\varepsilon }_{r}$=2.2, and a loss tangent tanδ = 0.0009. The ground-plane (GND) conductor has the same thickness of 0.035 mm. The antenna is excited by a metal pin inserted through a via-hole from the ground-plane. To ensure isolation from the ground-plane, the conductor surrounding the via-hole is etched away, exposing the dielectric substrate, which effectively forms a coplanar waveguide feed.

Figure 1d shows the antenna’s performance across a frequency band from 27.35 to 29.25 GHz, corresponding to a fractional bandwidth of 6.71%. The antenna’s average gain and radiation efficiency are measured as 1.4 dBi and 47%, respectively, as depicted in Fig. 1e, f.

#### Decoupling wall

**Fig. 2:**
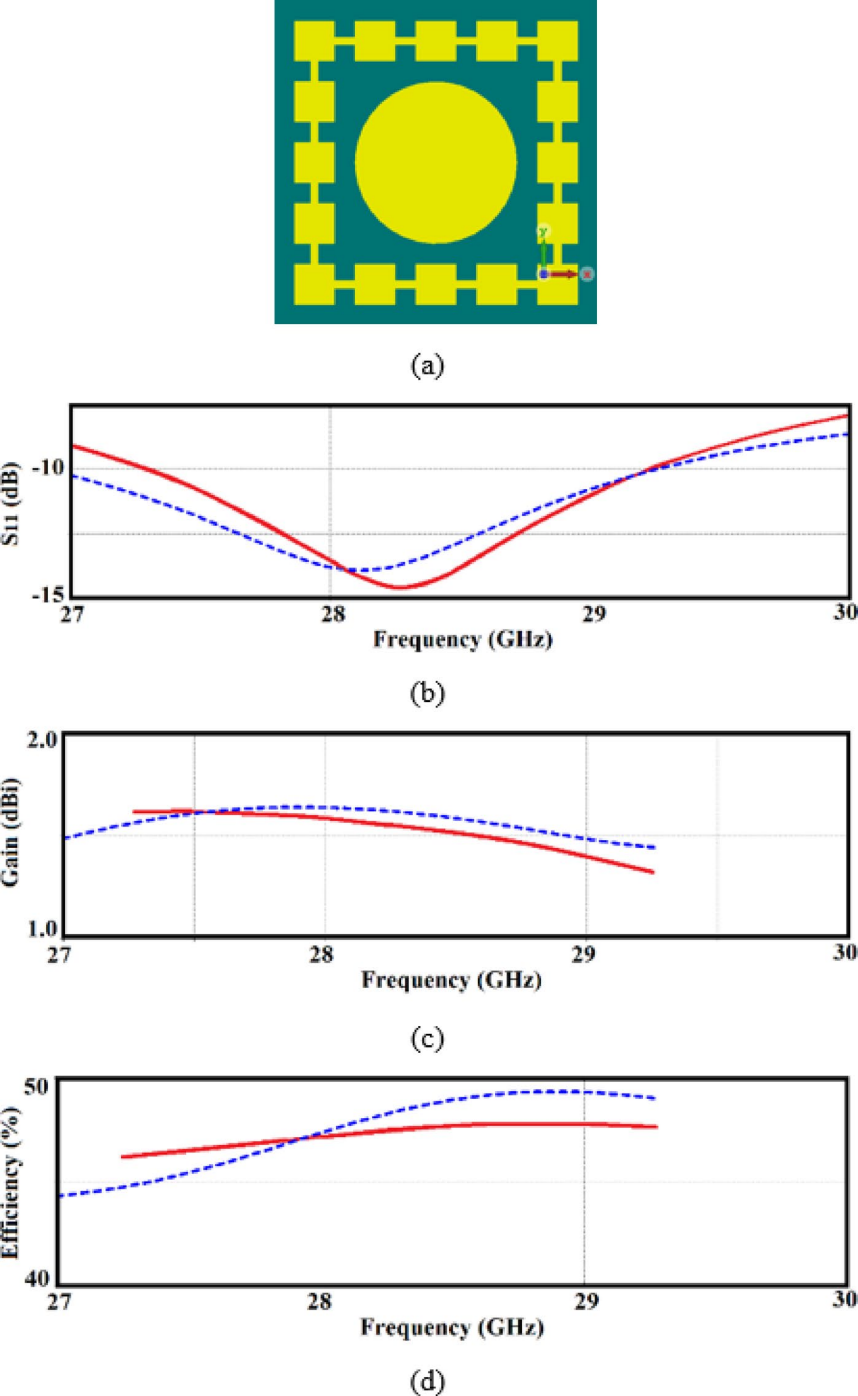
(**a**) Configuration of the proposed single-element antenna enclosed within decoupling wall made of a square periodic high-low impedance microstrip-line, (**b**) reflection coefficient (S_11_ < -10 dB) responses, (**c**) gain, and (**d**) radiation efficiency of both single-element antenna without and with the decoupling wall are represented by solid- and dashed-lines, respectively.

The proposed radiating element is designed for use in an antenna array, requiring a compact array size. However, closely spacing the radiating elements can result in strong coupling between them, degrading the array’s overall performance. To mitigate this issue, a transmission-line structure with periodically varying impedance, transitioning between high and low values, is incorporated around the radiating element, as shown in Fig. 2a.

The configuration of the transmission line, including the number, width, and spacing of the wide and thin sections, has been optimized to achieve high isolation in array applications. The width and length of the wide sections are set to 1 mm, with a 0.5 mm gap between them. This decoupling structure effectively suppresses surface waves, enhancing isolation between elements. Additionally, the design has minimal impact on the radiator’s bandwidth and radiation characteristics, as illustrated in Fig. 2b–d. The antenna has an overall size of only 8 × 8 × 0.67 mm^3^ which corresponds to 0.68*λ*_0_ × 0.68*λ*_0_ × 0.057*λ*_0_.

#### Radiator with multi-split ring slot

In this section, we draw inspiration from metamaterials to enhance the proposed antenna’s performance, including gain, impedance matching, bandwidth, and radiation efficiency. Metamaterials possess unique properties, such as negative permittivity and permeability, which are not typically found in natural materials ^31^. These properties enable precise control and manipulation of electromagnetic waves, making them particularly valuable for optimizing antenna performance at millimeter-wave frequencies used in 5G applications.

In the design, multiple ring slots on the patch act like a metasurface (MTS), which is a 2D equivalent of a 3D metamaterial. The structure modifies the effective aperture of the antenna, increasing its bandwidth and radiation properties. The key structural components are designed to emulate metamaterial parameters—series left-handed capacitances and shunt left-handed inductances. These are achieved by creating slots (capacitive elements) on the radiation patch and potentially using via-holes for inductive characteristics ^32^. However, for simplicity, the inductive components were excluded in this design, focusing solely on the capacitive slot elements.

**Fig. 3:**
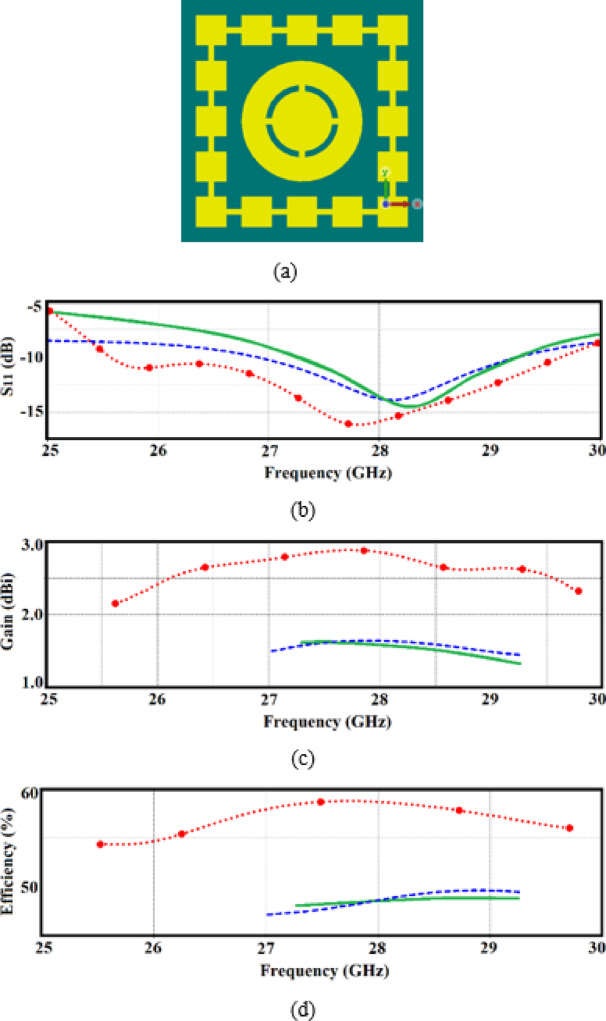
(**a**) Single-element antenna loaded with multi-split ring slot, (**b**) reflection coefficient response, (**c**) gain response, and (**d**) radiation efficiency. Original radiator represented by the green solid-line, radiator with decoupling wall represented by blue dashed-line, and radiator loaded with multi-split ring slot represented by red dotted-line.

By incorporating these multi-split ring slots, the antenna exhibits behaviours typical of metamaterials, such as improved control over electromagnetic wave propagation, which leads to enhanced performance metrics such as a significant boost in bandwidth, gain and radiation efficiency, as shown in Fig. 3.

The implementation of the MTS increases the effective aperture of the radiator, as demonstrated by the expanded impedance bandwidth where the reflection coefficient (S_11_) remains below − 10 dB, as shown in Fig. 3b. This enhancement is further underscored by improved impedance matching performance.

Without the multi-split ring loading, the radiator achieves a fractional bandwidth (FBW) of 6.71%, spanning from 27.35 to 29.25 GHz. However, the addition of the decoupling wall increases the FBW to 8.0%, covering a frequency range from 27 to 29.25 GHz. Notably, incorporating the multi-split ring slot significantly boosts the FBW to 14.82%, extending the operational range from 25.6 to 29.7 GHz.

Figure 3c and d further illustrate the improvements in radiation characteristics due to the slot loading. The average radiation gain and efficiency increase to 2.7 dBi and 57%, respectively, across the operational band of 25.6–29.7 GHz. This represents an improvement of 1.3 dBi and 10% compared to the configuration without slot loading, which exhibits an average gain of 1.4 dBi and an efficiency of 47% over the narrower range of 27–29.25 GHz.

### Antenna array

**Fig. 4:**
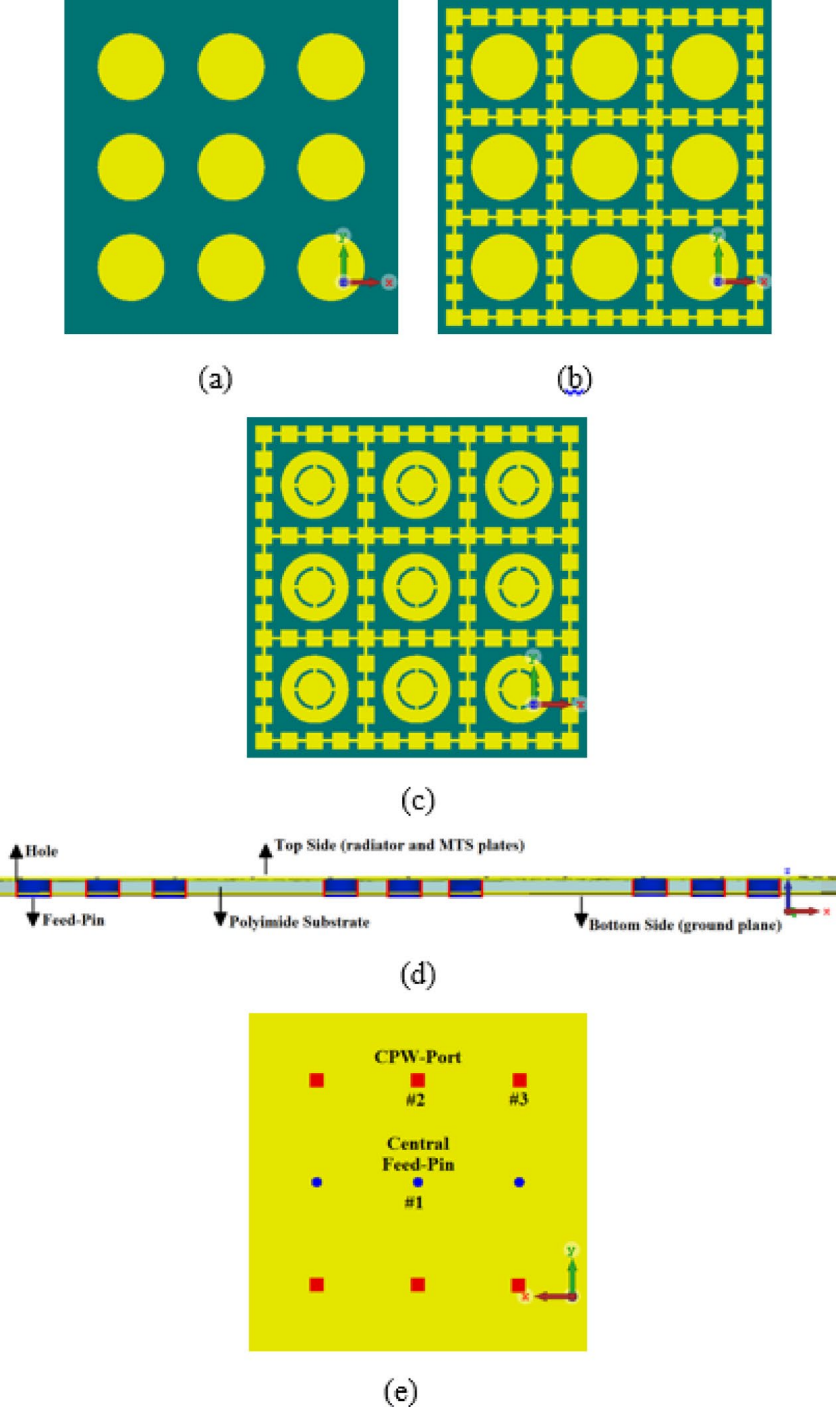
Steps in realizing the proposed antenna array, (**a**) topside view of the circular patch radiators, (**b**) the circular patch radiators enclosed inside decoupling walls, (**c**) the circular patch radiators loaded with multi-split ring slots contained inside decoupling walls, (**d**) cross-section view of the antenna array, and (**e**) bottom view of ground-plane indicating feed point positions.

The antenna array was constructed based on the radiator shown in Fig. 3a. The process of creating the 3 × 3 antenna array begins with the fabrication of circular radiators, whose diameters are determined by Eq. (1). These radiators are surrounded by decoupling walls and feature multi-split ring slots, as illustrated in Fig. 4. The radiators are excited by a metal pin inserted through a via-hole from the ground plane, making contact with the radiator from below. To isolate the pin from the ground plane, the conductor surrounding the via-hole is etched away, exposing the dielectric substrate underneath. This setup effectively creates a coplanar waveguide feed. The antenna array was fabricated on a Rogers RT5880 dielectric substrate with a thickness of 0.6 mm, a relative permittivity, ${\varepsilon }_{r}$= 2.2, and tanδ = 0.0009. The dimensions of the array are 20 × 20 × 0.67 mm^3^.

**Fig. 5:**
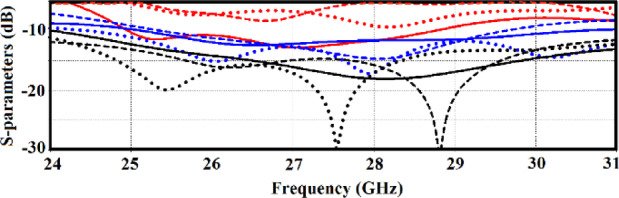
Comparison of the S-parameter responses of the antenna array based on (i) the radiators alone (red curve), (ii) the radiators with decoupling wall (blue curve), and (iii) the radiators with decoupling walls plus multi-split ring slots (black curve). S_11_, S_12_, and S_13_ curves are represented by solid-, dashed-, and dotted-lines, respectively.

Figure 5 illustrates the effect on the S-parameters of the array antenna under three different scenarios: (1) when the array consists only of radiators, (2) when decoupling walls are introduced, and (3) when multi-split ring slots are incorporated. The array ports are matched to 50 Ω loads. The results show that the array with only radiators operates over a frequency range of 25–28.7 GHz, with an average impedance matching of 12 dB and a fractional bandwidth (FBW) of 13.78%. The average port-to-port isolation between port #1 and port #2 (S_12_) and between port #1 and port #3 (S_13_) across this frequency band is measured at 5 dB and 6 dB, respectively.

When decoupling walls are introduced around the radiators, there is a 7% increase in fractional bandwidth and a 7.5 dB improvement in isolation. Further enhancement is observed when multi-split ring slots are added to the radiating elements. Compared to the radiators alone, this configuration results in an 11.67% increase in fractional bandwidth and a 12 dB improvement in isolation. Measured results confirm that the array design with multi-split ring slots effectively covers the 24–31 GHz frequency range, which is allocated for 5G New Radio (NR). A summary of these findings is presented in Table 1.

**Table 1:** S-parameters of the antenna arrays.

Parameters	S_11_ < –10 dB	Average isolation (S_12_)	Average isolation (S_13_)
Radiators alone	Freq. range: 25.0–28.7 GHz BW: 3.7 GHz FBW: 13.78%	–5 dB	–6 dB
With decoupling walls	Freq. range: 25.0–30.8 GHz BW: 5.8 GHz FBW: 20.78%	–12 dB	–14 dB
With multi-split ring slots	Freq. range: 24.0–31.0 GHz BW: 7.0 GHz FBW: 25.45%	–17 dB	–18 dB
Improvement compared with the radiators alone	BW: 3.3 GHz FBW: 11.67%	12 dB	12 dB

**Fig. 6:**
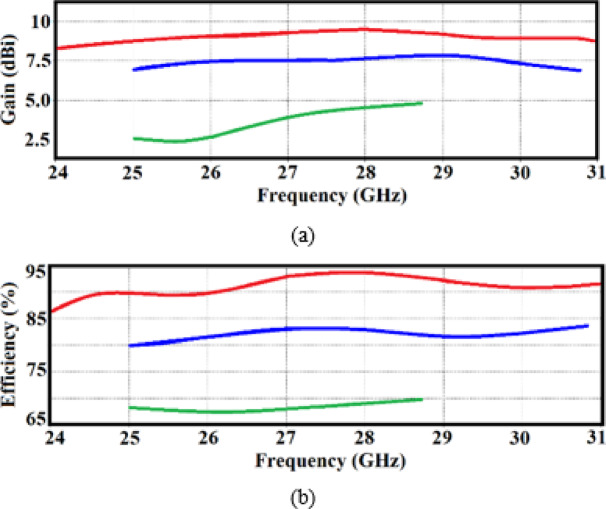
Radiation characteristics performances of the 3 × 3 antenna array, (**a**) gain, and (**b**) radiation efficiency. The radiators alone, with decoupling walls, and with decoupling walls plus multi-split ring slots are represented by green, blue, and red curves, respectively.

Figure 6a,b illustrate the radiation characteristics of the proposed 3 × 3 antenna array under three implementation scenarios: (i) using radiators alone, (ii) integrating radiators with decoupling walls, and (iii) incorporating radiators with decoupling walls and multi-split ring slots. In the first scenario, shown in Fig. 6a, the array with only radiators achieves an average antenna gain of 3.3 dBi within the 25–28.7 GHz range. In the second scenario, with the addition of decoupling walls, the average gain improves to 7.5 dBi over a frequency range of 25–30 GHz. Finally, in the third scenario, where both decoupling walls and multi-split ring slots are included, the array achieves a significantly higher average gain of 9 dBi across 25 GHz to 29 GHz—an improvement of 5.7 dB compared to the first case.

This increase in gain is attributed to the expanded antenna aperture resulting from the integration of multi-split ring slots, which enhance the antenna’s ability to capture and radiate electromagnetic waves more efficiently. The slots function as supplementary structures, focusing and directing electromagnetic energy in the desired direction. Consequently, the antenna array’s performance is greatly enhanced, improving both signal transmission and reception capabilities across the specified frequency range.

Figure 6b shows how the average radiation efficiency of the proposed 3 × 3 antenna array varies across the different implementation scenarios. In case (i), where only radiators are used, the average radiation efficiency is 67% over the frequency range of 25–28.7 GHz. In case (ii), with the addition of decoupling walls, the efficiency improves to 83% across 25–30 GHz. In case (iii), where both decoupling walls and multi-split ring slots are incorporated, the efficiency reaches 92% between 25 and 29 GHz—an improvement of 25% compared to the first scenario.

This substantial increase in radiation efficiency is attributed to the combined effects of the decoupling walls and multi-split ring slots, which work together to reduce losses and impedance mismatches. These enhancements optimize the antenna’s ability to convert input power into radiated electromagnetic energy, thereby improving overall performance across the frequency spectrum.

### Experimental results and discussions

**Fig. 7:**
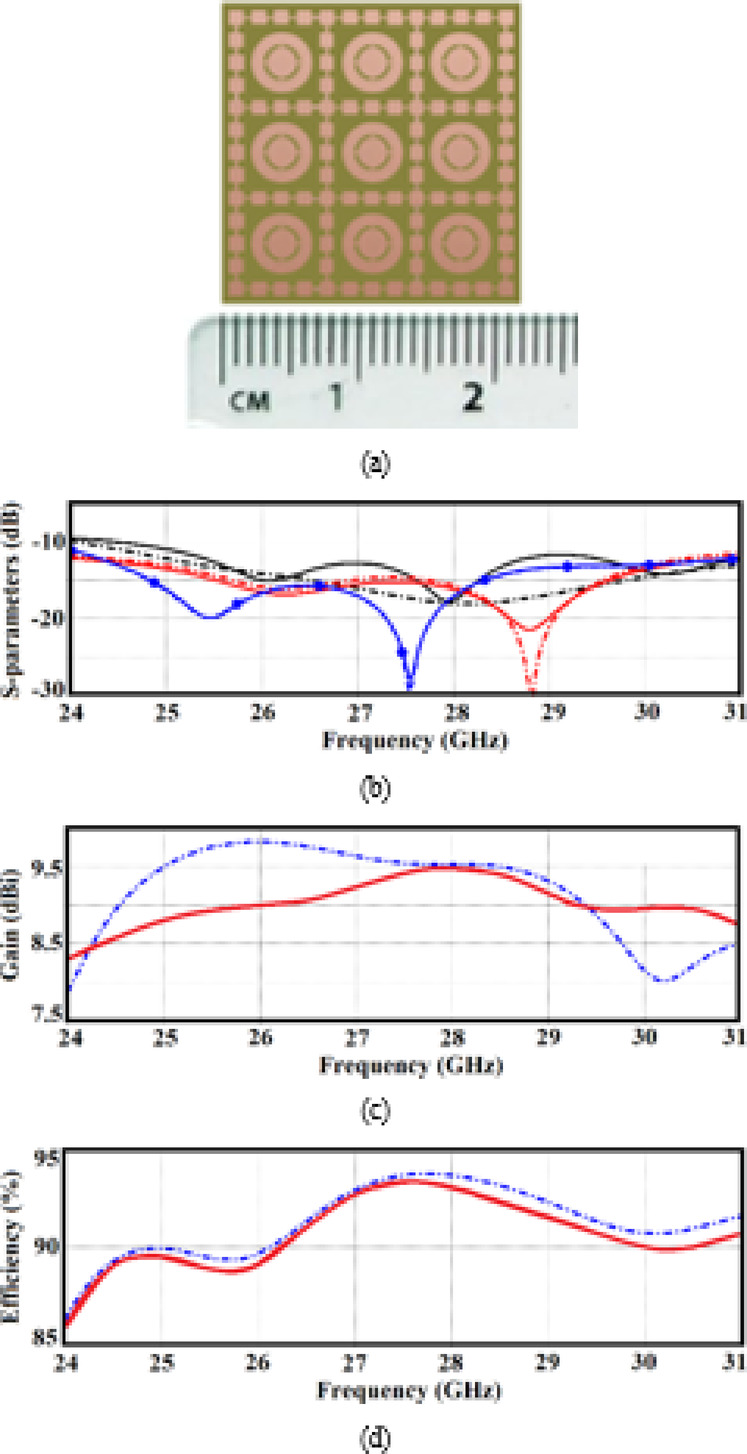
(**a**) Fabricated prototype of the proposed 3 × 3 antenna array, (**b**) S-parameters, S_11_, S_12_, and S_13_ are represented by black, red, and blue curves, respectively, (**c**) gain, and (**d**) radiation efficiency. Dashed- and solid-lines represent the simulated and measured results, respectively.

The 5G millimeter-wave band has driven demand for low-profile array antennas due to their ability to accelerate data transmission, increase network capacity, and improve link reliability. In response, we propose a 3 × 3 antenna array designed to meet these requirements. The array was fabricated on a Rogers RT5880 substrate, with a thickness of 0.6 mm, a dielectric constant ${\varepsilon }_{r}$ =2.2, and a dielectric loss tangent tan δ = 0.0009. Photolithography techniques with a precision of 0.05 mm were used in the fabrication process, as shown in Fig. 7a. Each radiator is excited through a 0.3 mm radius via-hole from beneath the substrate. The array’s performance was tested using a Vector Network Analyzer (VNA) (Agilent Technologies E8364B model).

The simulated and measured reflection and transmission coefficients are presented in Fig. 7b. Due to the symmetrical layout of the array, only S-parameters for ports #1 to #3 are shown. Each port’s feedline was matched to a 50 Ω load. The measured results confirm excellent impedance matching, with ∣S_11_∣ consistently below -10 dB across the entire 5G mm-wave band from 24 to 31 GHz, corresponding to a fractional bandwidth of 25.45%. This range includes the 26/28 GHz band, which is widely adopted or under consideration for 5G New Radio (NR) in many countries ^13^. The array also demonstrates high isolation performance, with isolation between adjacent elements (S_12_) and diagonal elements (S_13_) exceeding 17 dB, thanks to the decoupling walls. The results show strong agreement between simulated and measured data, with any discrepancies falling within acceptable manufacturing tolerances.

Far-field radiation measurements were conducted in an anechoic chamber. A standard gain horn antenna served as the transmitter, while the prototype antenna acted as the receiver. The antenna under test was rotated to capture variations in radiation intensity across different angles. Due to the planar structure of the array, no misalignment issues were encountered. Figure 7c illustrates both the simulated and measured radiation gain responses. The gain increases from 8.4 dBi at 24 GHz to 9.5 dBi at 28 GHz. Over the 24– 31 GHz frequency range, the average gain remains steady at 9.0 dBi, demonstrating minimal variation and meeting the high-gain requirements for 5G mm-wave communications.

Simulated and measured radiation efficiency plots are shown in Fig. 7d, again demonstrating good agreement. The efficiency ranges from 86.5% at 24 GHz to 93.5% near 28 GHz, with an average of 91.5% across the entire 24–31 GHz band. This impressive performance is largely attributed to the integration of multi-split ring slots, which enhance radiation efficiency throughout the operating frequency range.

**Fig. 8:**
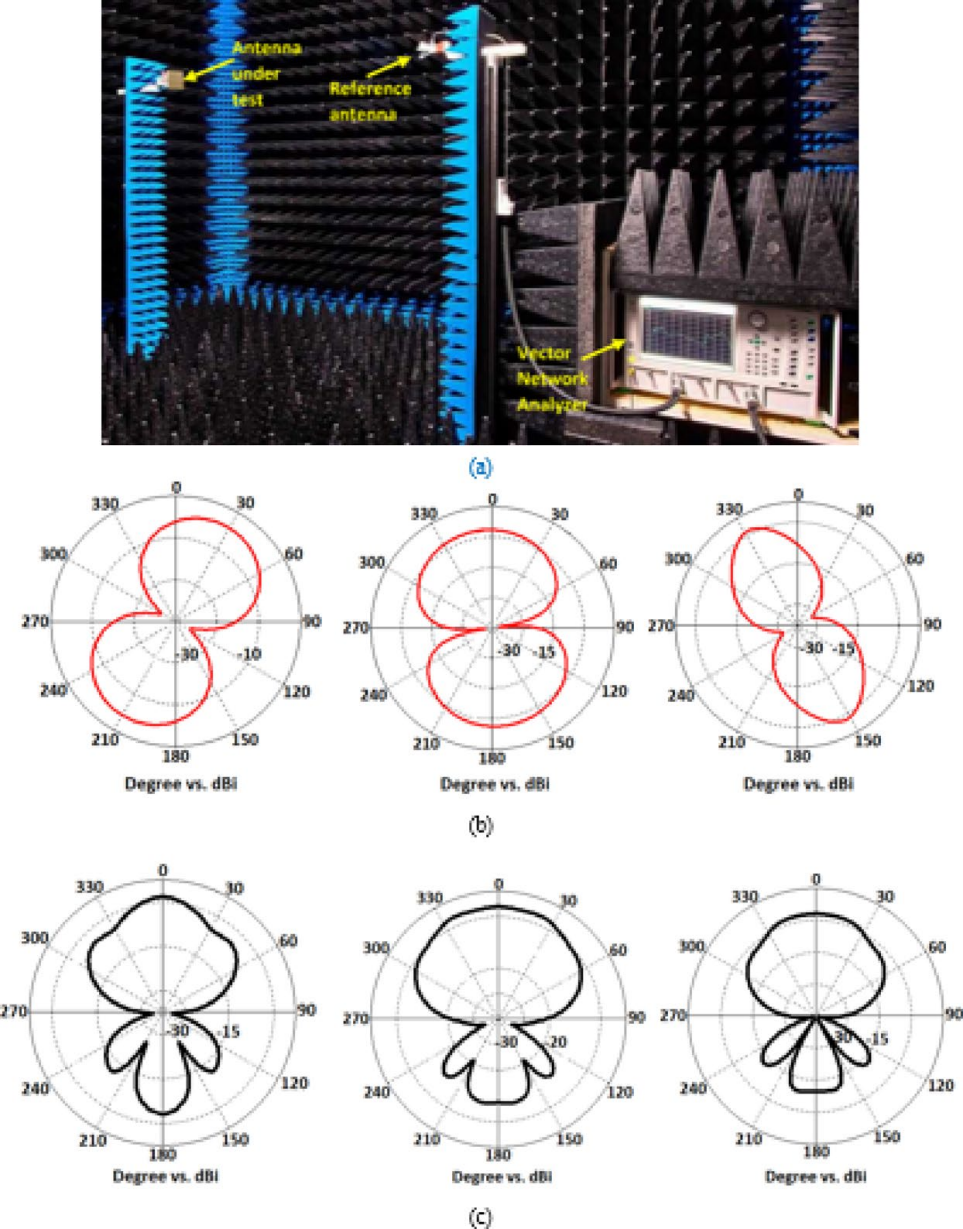
Measured radiation patterns of the proposed 3 × 3 antenna array at spot frequencies of 24 GHz, 28 GHz & 31 GHz (from left to right), (**a**) measurement set-up, (**b**) E-plane, and (**c**) H-plane.

The measurement setup and radiation characteristics of the 3 × 3 antenna array is shown in Fig. 8. The reference antenna was connected to the Vector Network Analyzer (VNA) as the receiver, while the antenna under test (AUT) was connected to a signal source. The radiated power from the AUT was measured at various angles to capture its radiation pattern. The results in Fig. 8 indicate that both the E-plane and H-plane patterns fall within the antenna’s operational range.

In the E-plane, the antenna exhibits bi-directional broadside radiation with patterns resembling a dumbbell shape. At 24 GHz, the radiation is tilted by + 30°, while at 28 GHz it is aligned directly at 0°, and at 31 GHz it tilts by -30°. Additionally, the 3 dB beamwidth narrows at 31 GHz. In the H-plane, the antenna predominantly radiates unidirectionally, although a significant back-lobe is present.

Further analysis of the E-plane reveals polarization behavior. At 24 GHz, left-hand circular polarization (LHCP) is minimal compared to right-hand circular polarization (RHCP) at the broadside (θ = 0°). At 31 GHz, this behavior is reversed, with LHCP dominant at the broadside. At the center frequency of 28 GHz, both LHCP and RHCP are present at the broadside. Moreover, the E-plane patterns rotate counterclockwise as the frequency increases from 24 to 31 GHz, indicating the presence of both RHCP and LHCP components.

## Performance comparison with prior arts

The proposed single-layer antenna array is compared with arrays from current literature, focusing on key parameters such as height profile, frequency bandwidth, operating frequency, radiation gain, number of layers, air gap, and array functionality. The comparison results, presented in Table 2, highlight several advantages of the proposed array, including a compact height profile, wide bandwidth, comparable center frequency operation, strong gain performance, and full array functionality.

**Table 2:** State-of-the-art comparison.

Antennas	Applied technique	Height profile (${\lambda }_{0}$)	BW (%)	${f}_{o}$(GHz)	LP/CP	Max. Gain (dBi)	Layers	Air gap	Array functionality
^13^	Patch + MTS	0.04	23.4	27.5	CP	11	1	$\times$	$\surd$
^21^	Patch + MTS	0.093	34.7	27.5	CP	11	2	$\times$	$\times$
^33^	Patch + MTS	0.04	18	5.7	CP	8.4	1	$\times$	$\times$
^34^	Patch + Parasitic	0.01	Not given	2.5	CP	Not given	1	$\times$	$\times$
^35^	Patch + Parasitic	0.016	6	3.05	CP	2.7	1	$\times$	$\times$
^36^	Patch + DGS	0.07	14.6	28	CP	8.3	1	$\times$	$\surd$
^37^	Patch + EBG	Not given	3	24	LP	6	1	$\times$	$\surd$
^38^	Patch + DGS	0.13	15.6	25.2	LP	8.7	1	$\times$	$\surd$
^39^	Patch + DGS	0.06	39	31	LP	10.6	1	$\times$	$\surd$
^40^	Patch + Superstrate	0.35	19	30	LP	8	3	$\surd$	$\surd$
^41^	Patch + Superstrate	0.7	27.6	28	CP	14.1	2	$\surd$	$\surd$
^42^	Vivaldi Antenna	0.5	6	28/38	CP	8	1	$\times$	$\surd$
^43^	Yagi Uda + EBG	0.36	19	29	CP/LP	11.9	3	$\times$	$\times$
^44^	Dipole + SRR	0.27	17.2	29.5	CP	11.9	2	$\surd$	$\times$
^45^	Dielectric Resonator	0.12	34	25	CP	8.1	4	$\times$	$\times$
^46^	Cross Dipole	0.15	8	28	CP	2.2	1	$\times$	$\times$
This work	MTS + Ring Slot	0.025	25.45	26/28	CP	9.5	1	$\times$	$\surd$

MTS, DGS, EBG, SRR, ${\lambda }_{0}$, BW, ${f}_{o}$, CP, and LP stand for 'metasurface,' 'defected ground structure,' 'electromagnetic bandgap,' 'split-ring resonator,' free-space wavelength at the center frequency, bandwidth, operating frequency, circular polarization, and linear polarization, respectively.

In contrast, antennas described in ^33–35^ offer benefits such as single-layer construction and circular polarization (CP) radiation but suffer from narrow bandwidths, lack of array support, and lower gain despite operating at lower frequencies. The superstrate antenna in ^41^ performs well in terms of array functionality, bandwidth, and gain but has drawbacks related to its multilayer structure, complex design, and the inclusion of an air gap. Similarly, the CP array in ^40^ achieves only 8 dBi gain and a limited CP bandwidth of 3%. Furthermore, CP antennas in ^43–46^ do not support array configurations.

Table 3 provides additional comparisons, including the center-to-center spacing between radiating elements and isolation performance. The proposed array features the narrowest spacing and achieves 17 dB isolation across its operational bandwidth. This high isolation is attributed to the decoupling walls surrounding each radiating element. Additionally, the increased aperture, resulting from the embedded multi-split ring slot in the disk-shaped patch radiator, contributes to the enhanced gain.

**Table 3:** Array feature comparison.

Refs.	Center-to-center spacing (${\lambda }_{o}$)	Min. Isolation (dB)
^13^	0.36	30
^36^	Not given	17
^37^	0.4	37
^38^	0.84	16.2
^39^	1.06	22
^40^	0.5	20
^41^	0.6	25
^42^	Not given	16
This work	0.16	17

## Conclusion

A novel wideband circularly polarized 3 × 3 antenna array has been successfully demonstrated for 5G millimeter-wave (mm-wave) applications, meeting the performance requirements of smart devices and sensors. The array, designed on a single-layer dielectric substrate, features compact circular disk-shaped radiating elements, each isolated by a decoupling wall to prevent performance degradation. To optimize the aperture, a metasurface-inspired technique was implemented, embedding multi-split ring slots on each radiating element, which significantly enhances the array’s performance.

Each element is fed through a metallic pin via a hole in the ground-plane, connected to coplanar waveguide (CPW) ports. Experimental results confirm excellent performance across a wide frequency range of 24 to 31 GHz, achieving a fractional bandwidth of 25.45%. The array demonstrates key performance metrics, including an average isolation of over 17 dB between radiating components and average radiation gain and efficiency of 9.0 dBi and 91.5%, respectively.

Compared to alternative antenna array designs, the proposed solution offers notable advantages in simplicity and cost-effectiveness. Unlike more complex arrays that require intricate manufacturing or specialized materials, this design prioritizes practicality and affordability without compromising performance.

## Data Availability

All data generated or analyzed during this study are included in this published article.
